# Pannexin1: Role as a Sensor to Injury Is Attenuated in Pretype 2 Corneal Diabetic Epithelium

**DOI:** 10.1155/2021/4793338

**Published:** 2021-07-13

**Authors:** Garrett Rhodes, Kristen L. Segars, Yoonjoo K. Lee, Audrey E. K. Hutcheon, Celeste B. Rich, Vickery Trinkaus-Randall

**Affiliations:** ^1^Department of Ophthalmology, Boston University School of Medicine, Boston, Massachusetts 02118, USA; ^2^Department of Pharmacology, Boston University School of Medicine, Boston, Massachusetts 02118, USA; ^3^Schepens Eye Research Institute of Mass Eye and Ear, Department of Ophthalmology, Harvard Medical School, 20 Staniford Street, Boston, MA 02114, USA; ^4^Department of Biochemistry, Boston University School of Medicine, Boston, Massachusetts 02118, USA

## Abstract

Epithelial wound healing is essential to repair the corneal barrier function after injury and requires coordinated epithelial sheet movement over the wounded region. The presence and role of pannexin1 on multilayered epithelial sheet migration was examined in unwounded and wounded corneal epithelium from C57BL/6J (B6) control and diet-induced obese (DiO) mice, a pretype 2 diabetic model. We hypothesize that pannexin1 is dysregulated, and the interaction of two ion-channel proteins (P2X7 and pannexin1) is altered in pretype 2 diabetic tissue. Pannexin1 was found to be present along cell borders in unwounded tissue, and no significant difference was observed between DiO and B6 control. However, an epithelial debridement induced a striking difference in pannexin1 localization. The B6 control epithelium displayed intense staining near the leading edge, which is the region where calcium mobilization was detected, whereas the staining in the DiO corneal epithelium was diffuse and lacked distinct gradation in intensity back from the leading edge. Cells distal to the wound in the DiO tissue were irregular in shape, and the morphology was similar to that of epithelium inhibited with 10Panx, a pannexin1 inhibitor. Pannexin1 inhibition reduced mobilization of calcium between cells near the leading edge, and MATLAB scripts revealed a reduction in cell-cell communication that was also detected in cultured cells. Proximity ligation was performed to determine if P2X7 and pannexin1 interaction was a necessary component of motility and communication. While there was no significant difference in the interaction in unwounded DiO and B6 control corneal epithelium, there was significantly less interaction in the wounded DiO corneas both near the wound and back from the edge. The results demonstrate that pannexin1 contributes to the healing response, and P2X7 and pannexin1 coordination may be a required component of cell-cell communication and an underlying reason for the lack of pathologic tissue migration.

## 1. Introduction

Obesity is a major risk factor for the development of type 2 diabetes. The cornea is an excellent organ to examine the effects of diet on wound healing, as it is a relatively simple avascular tissue that is oxygenated via diffusion. Corneal epithelial repair requires a number of synchronized events that include, but are not limited to, cell motility, cell-cell communication, matrix deposition, and tissue remodeling [1, 2]. In diabetes, the apical corneal epithelium and its tight junctions become compromised, and large molecules and pathogens can penetrate into the epithelium. This impaired function appears to be exacerbated in patients with elevated HbA1c levels [3–5] and enhances the risk for delayed wound healing after injury, epithelial fragility, and possibly decreased corneal epithelial stiffness [6–8].

One of the earliest demonstrations of injury response was performed by live-cell imaging of rabbit corneal epithelial cells. In this study, it was observed that the injury-induced release of nucleotides triggered a transient calcium (Ca^2+^) mobilization near the wound site that crossed acellular zones, indicating that it was independent of gap junctions [9]. In addition to the initial release of ATP, factors such as EGF, cytokines, and other nucleotides were released from tears, activated growth factor receptors, and other proteins [10, 11]. This ATP binds and activates a large family of transmembrane purinoreceptors (P2Y and P2X receptors) in the cornea resulting in downstream signaling [9, 11, 12]. The G-protein P2Y receptors cause an increase in intracellular Ca^2+^, while the P2X receptors are ion channels and gate calcium and other ions [13, 14]. One P2X receptor, P2X7, is often considered to be a pain receptor, is expressed by both corneal epithelium and nerves [15, 16], and mediates cell migration [17]. Under normal physiological conditions, P2X7 localization is intense in the epithelial cells near the leading edge of the wound; however, once the edges of the wound have made contact and the epithelium restratifies, its localization realigns along the cell borders [17]. In vitro studies revealed that activation of the P2X7 receptor elicits a unique Ca^2+^ mobilization profile that travels throughout the epithelial sheet in a predictive cyclic pattern displaying cell-cell communication not through gap junctions but through pannexin channels [18].

Studies on diabetic corneal epithelium revealed a significant increase (4-7-fold) in P2X7 mRNA compared to age matched individuals [15, 17]. In addition, there is a report that patients with type 2 diabetes had increased P2X7 in peripheral blood mononuclear cells compared to control individuals [19]. Previous studies on corneal epithelium from a pretype 2 diabetic obese murine model demonstrated that there was a loss of sensory epithelial nerves and a change in P2X7 localization in the epithelium and stroma after corneal abrasions [6]. Furthermore, investigations have shown that interaction of P2X7 and pannexin1, another ion channel, was present in cultured corneal epithelial studies, while other studies showed that P2X7 knockout mice displayed reduced pannexin1 [18, 20, 21]. These data indicate that both pannexin1 and P2X7 may act as sensing proteins near the leading edge of the wound.

The pannexins belong to a family of large pore proteins that transport and release intracellular ATP into the interstitial space through a single membrane channel, and in so doing, play a number of roles in signaling, inflammation, and cytoskeletal reorganization [22, 23]. Researchers have hypothesized that ATP moves through pannexin channels and activates P2X receptors in a feed-forward pattern [24]. Recently, we detected pannexin1 near the leading edge of the wound in cultured epithelial cells and demonstrated that P2X7 receptor activation was abrogated by pannexin1 inhibition [18]. Taken together, these results suggest that epithelial cells display a complex repertoire of communication between pannexin1 and P2X7.

The major goals of this study were to determine if there was a difference in the localization of pannexin1 and the association between P2X7 and pannexin1 in C57BL/6J (B6) control and pretype 2 diabetic (DiO) murine corneal epithelium. We hypothesize that pannexin1 facilitates the healing process through its localization near the leading edge, and the subsequent movement of ATP through its channels activates P2X7 receptors, thus promoting migration. While pannexin1 localization was similar in unwounded corneal epithelium from both tissues, there were striking differences after injury. After 2 hours, pannexin1 in the region adjacent to the leading edge was intense in B6 control and diffuse in DiO; however, after 20 hours, this response was less intense in both the B6 control and DiO tissue. When pannexin1 was inhibited, there was a 50% decrease in cell-cell communication, compared to B6 control. Furthermore, proximity ligation assays (PLA) demonstrated that while P2X7 and pannexin1 displayed a similar level of interaction in unwounded corneas, there was a significant decrease in association in the wounded DiO corneal epithelium. The potential synergistic relationship and physical association indicate that pannexin1 may be a potential target for pharmacological intervention of wounds.

## 2. Materials and Methods

### 2.1. Antibodies and Inhibitors

For immunohistochemistry, anti-pannexin1 polyclonal rabbit antibody directed against pannexin1 (Cat. #ACC-234) and anti-P2X7 polyclonal rabbit antibody targeting the P2X7 receptor (Cat. #APR-004) were purchased from Alomone Labs (Jerusalem, Israel). For proximity ligation assay, anti-pannexin1 monoclonal mouse antibody directed against pannexin1 (Cat. #SC-515941) was purchased from Santa Cruz Biotechnology (Dallas, TX), and anti-P2X7 polyclonal rabbit antibody targeting the extracellular domain of P2X7 receptors (Cat. #APR-008) was purchased from Alomone Labs. 10Panx, a pannexin1 mimetic inhibitory peptide (Cat. #3348), and its scrambled peptide were purchased from Tocris Biosciences (Minneapolis, MN). Alexa Fluor-conjugated Plus 488 donkey anti-rabbit and mouse IgG secondary antibodies and rhodamine-phalloidin were purchased from Invitrogen (Carlsbad, CA).

### 2.2. Tissue Preparation and Organ Culture

All mice were purchased from Jackson Laboratory (The Jackson Laboratory; Bar Harbor, ME), and research protocols conformed to the standards of the Association for Research in Vision and Ophthalmology for the Use of Animals in Ophthalmic Care and Vision Research, as well as the Boston University IACUC. The C57BL/6J mice were fed either a control diet (B6 control) or a high fat diet to induce obesity (DiO) (Jackson Laboratory): Control Diet D12450B (10 kcal% fat, 3.8 kcal/g) and High Fat Diet D12492 (60 kcal% fat, 5.2 kcal/g) [6].

To examine localization of P2X7 and pannexin1 in response to injury, 1.5 mm debridement wounds were performed on both B6 control and DiO mice, as previously described [6, 17, 18]. After debridement, the mice were euthanized, the eyes enucleated, and placed in organ culture [12, 17], and incubated in serum-free Keratinocyte medium (Invitrogen Carlsbad, CA) containing 100 *μ*g/mL penicillin and 100 *μ*g/mL streptomycin for 2, 8, or 20 hours at 37°C and 5% CO_2_. A minimum of 3 eyes per time point per condition was analyzed.

### 2.3. Immunofluorescence

After each time point, the globes were fixed in freshly prepared 4% paraformaldehyde in phosphate-buffered saline (PBS), pH 7.2, for 30 minutes at room temperature (RT). Immunofluorescent staining was performed [17] by permeabilizing and blocking the tissue in blocking solution (PBS (93% *v*/*v*), Triton X-100 (10% *v*/*v*), BSA (5% *v*/*v*)) for one hour (hr) at RT. The tissue was incubated in primary antibody diluted in blocking solution overnight at 4°C (dilution will be described with each experiment) and washed with PBS. Alexa Fluor-conjugated 488 secondary antibodies (Invitrogen, Carlsbad, CA) were used at a dilution of 1 : 300 in blocking solution for 1 hr at RT prior to being washed again with PBS. The tissue was counter-stained with rhodamine-conjugated phalloidin (Invitrogen, 1 : 50) to visualize F-actin. Cells or tissues were mounted using VectaSHIELD containing 4′,6-diamidino-2-phenylindole (DAPI) (Vector Labs, Burlingame, CA) to visualize cell nuclei. The primary antibody was excluded from the secondary control tissue. Following staining, the corneas were imaged using confocal microscopy.

### 2.4. Confocal Microscopy for Immunofluorescence

The tissue was prepared by slicing the cornea into radial sections and cutting butterfly slits into the scleral rim in order to flatten the tissue for a uniform image [17]. Tissues were sandwiched between a p35 MatTek tissue culture dish (MatTek; Ashland, MA) and a glass coverslip, and imaged enface from the apical epithelium toward the stroma on a Zeiss LSM 700 (Zeiss, Thornwood, NY) confocal microscope. Fluorescent gain levels were set using secondary control samples, with the pinhole size maintained at 1 Airy unit. The settings and objectives were not changed across all images in order to eliminate changes in data due to varying confocal settings. Each region was collected as a *Z*-stack in 1 *μ*m steps and included the apical and basal cells.

### 2.5. Proximity Ligation Assay (PLA)

Detection of proteins by proximity ligation assay (PLA) was performed using an anti-pannexin1 monoclonal mouse antibody and an anti-P2X7 polyclonal rabbit antibody that targets the extracellular domain of the P2X7 receptor. Detection was carried out using Duolink® reagents (Sigma-Aldrich, St. Louis, MO) according to the manufacturer's Duolink® PLA Fluorescence Protocol with slight modifications. The donkey anti-mouse MINUS (DUO92004) and donkey anti-rabbit PLUS (DUO92002) PLA probes, along with Duolink® blocking solution, antibody diluent, ligation buffer, amplification buffer, and specific wash buffers, were used in the assay. Tissue sections were incubated in blocking buffer for 60 minutes at 37°C in a preheated humidity chamber. Antibodies were diluted 1 : 100 (anti-pannexin1) and 1 : 300 (anti-P2X7) in antibody diluent, applied to tissue, incubated for 2 hrs at RT, and then washed in 1x Wash Buffer A at RT. PLUS and MINUS probes were diluted in antibody diluent mixture, and sections were incubated for 1 hr at 37°C in the heated humidity chamber. Sections were washed in Wash Buffer A at RT. The ligation buffer was mixed in high purity water, and ligase was added and incubated for 30 minutes at 37°C in a heated humidity chamber. Sections were washed in Wash Buffer A at RT. Sections were incubated in amplification buffer containing polymerase for 100 minutes at 37°C in a heated humidity chamber. Sections were washed in Wash Buffer B at RT and then with 0.01x Wash Buffer B. The tissue was imaged on the Zeiss Axiovert LSM 700.

### 2.6. Analysis of Proximity Ligation

Images were taken both near the leading edge and contiguous regions back from the edge using both the tiling function and optical slices. Analysis was conducted with ImageJ and CellProfiler (Broad Institute, Cambridge MA) to determine if DNA ligation occurred by assessing fluorescence, which only occurred if the PLUS and MINUS PLA probes were within 40 nm of each other [25]. To analyze for puncta, the images were imported into ImageJ; an initial thresholding was performed to separate puncta from background noise, and the file was exported into CellProfiler. The Spot Detection pipeline was used to count puncta. The size of an average puncta was determined using the measure tool in ImageJ and refined in CellProfiler. For consistency, the same analytical parameters were used for all images. Statistical analysis was performed using GraphPad Prism 5.

### 2.7. Calcium (Ca^2+^) Mobilization

Ca^2+^ mobilization experiments were performed on ex vivo mouse corneas [18]. In brief, the corneas were preincubated in Cal-520 AM and CellMask™ Deep Red (Thermo Fisher, Waltham, MA) for 30 minutes at 37°C and 5% CO_2_. Cal-520 AM was used at 1 : 100, and CellMask Deep Red was used at 1 : 10,000, and the final concentration of DMSO and pluronic acid was 1% (*v*/*v*) and 20% (*w*/*v*), respectively. Excess dye was removed, and the corneas were mounted in polyethylene glycol on silanized glass bottom dishes [26]. For in vitro imaging, human corneal epithelial (HCE) cells [17] were cultured to confluence on glass bottom dishes and preloaded with the calcium indicator dye. Images were collected every 5 seconds for 45 minutes on a Zeiss Axiovert LSM 880 confocal microscope (Carl Zeiss, White Plains, NY) utilizing the FAST module and AIRYScan.

### 2.8. Modeling of Ca^2+^ Waves

Image analysis was performed using FIJI/ImageJ (NIH, Bethesda, MD) and MATLAB scripts (MATLAB, MathWorks, Inc.) that were written specifically for the analysis of Ca^2+^ waves, as previously described [12, 18, 27]. In brief, videos from each experiment were exported as TIF or AVI format, imported into the custom MATLAB scripts, and analyzed for Ca^2+^ responses based on cell population (individual cells or groups of cells). These specific MATLAB scripts enabled the evaluation of the spatiotemporal and cell-cell communication of these cell populations by identifying Ca^2+^ events, generating an event kymograph, and calculating the probability of neighboring cells having a Ca^2+^ event that was induced within 10 frames after an established Ca^2+^ event occurred in a given cell. An automated computer program developed in the lab marked each cell's position and tracked the cells by calculating each *X* and *Y* centroid location for every registered individual area from the reference frame. To detect these events, the starting frame was chosen manually. Additional scripts were employed to correct for slight movement or drift over the frames.

### 2.9. Statistical Analysis

Values were obtained by taking the mean ± standard error of the mean (SEM) from at least three independent experiments. Statistical significance was determined by unpaired, one-tailed Student's *t*-test or one or two-way ANOVA with appropriate post hoc tests using GraphPad Prism 5 (GraphPad Software, San Diego, CA) and R studio (RStudio, Inc., Boston, MA).

## 3. Results and Discussion

### 3.1. Expression and Localization of Pannexin1

Pannexins belong to a family of proteins that release intracellular ATP through a single membrane channel, which then activate purinoreceptors, specifically the ion channel receptor, P2X7 [24]. The pannexin1 channel has a number of roles in signaling, inflammation, and cytoskeletal reorganization caused by mechanical deformation [22, 23]. Recently, Lee et al. [18] showed in vitro that inhibition of pannexin1 delayed wound healing, and we attributed this to aberrant directional motility. In addition, there was a decrease in cell-cell communication. Previously, we showed that there was a change in localization of P2X7 after an epithelial debridement in B6 control mice. After wound closure and epithelial stratification, this localization returned to that observed in unwounded tissue [17]. In contrast, there was a difference in localization of P2X7 in DiO corneas after wounding [6]. Together, these data led us to speculate that both P2X7 and pannexin1 may act as sensors at the leading edge of a wound; however, in tissue where wound healing is compromised, these sensors may not occur. Furthermore, it is not known if the association between P2X7 and pannexin1 is necessary for cell-cell communication and if this interaction is detected in obese or diabetic tissue.

**Figure 1:**
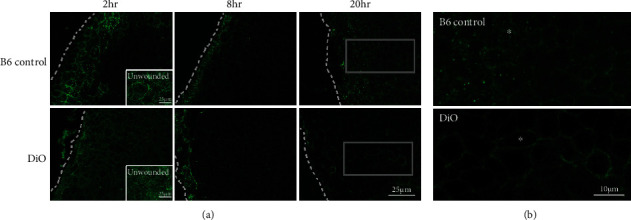
Pannexin1 localization in B6 control and DiO corneal epithelium. (a) Representative confocal immunofluorescent images of pannexin1 in unwounded and wounded corneas: 2, 8, and 20 hrs postepithelial debridement. Dotted line indicates the leading edge. Bar equals 25 microns. (b) Enlarged immunofluorescent images of the rectangular boxes in (a) (20 hrs), which show the cell morphology back from the leading edge. Asterisks (^∗^) mark representative cells in both B6 control and DiO ~6 cells back from the leading edge. Note the elongation of the ^∗^ cell in DiO. Bar equals 10 microns. *N* is a minimum of 3 independent experiments.

To further explore this relationship, the localization of pannexin1 was examined in epithelium before and after a debridement wound from B6 control and pretype 2 diabetic (DiO) mice (Figure 1). In both B6 control and DiO unwounded epithelium, pannexin1 was detected along the cell borders (Figure 1(a) (inset)), with the DiO tissue displaying a more extensive cytoplasmic pannexin1 staining. Two hours postdebridement, pannexin1 staining in the B6 control epithelium was intense along the cell borders near the leading edge and about 5-6 rows back (Figure 1(a)). In contrast, pannexin1 in the DiO tissue was diffuse and lacked the intensity detected in the B6 control (Figure 1(a)). At 8 hrs, the area of intense staining along the cell borders in the B6 control tissue decreased to only two rows immediately at the leading edge (dotted line). Interestingly, the staining pattern and intensity in the 8 hr DiO tissue were similar to B6 control. In addition, the leading edge in the DiO tissue was consistently more ragged, as they were more fragile upon manipulation. By 20 hrs, pannexin1 was present along the epithelial cell borders in both DiO and B6 control; however, punctate staining near the leading edge was prominent in the B6 control. We speculate that this localization is indicative of endocytosis in response to elevated ATP. Distal to the leading edge at 20 hrs (Figure 1(a), outline), the prototypical basal epithelial cobblestone shape was present in the B6 control tissue, while the cells were more elongated in the DiO (Figure 1(a), outline; enlarged in Figure 1(b) (asterisk)). We speculate that the elongated morphology was due to a change in mechanical force, which altered the cytoskeletal structure of the cells. This hypothesis was supported by the detection of a similar stretched epithelial phenotype in live-cell movies of cultured epithelial cells treated with 10Panx, a specific inhibitor of pannexin1 [18].

We hypothesize that injury induces a distinct phenotype, and the DiO tissue does not appear to induce the proper signal to activate remodeling. One potential reason for this may be that the compliancy of the diabetic epithelium is reduced causing a change in the mechanical deformation as cells move, which may alter the pannexin1-mediated ATP release and the activation of the P2X7 receptor seen in other tissues [23]. Supporting this speculation is experimental data demonstrating that DiO epithelium is significantly softer than the B6 control [8] and preliminary data indicating that diabetic epithelium is softer than DiO tissue.

### 3.2. Inhibition of Pannexin1 Mediates Actin Reorganization and Cell-Cell Communication

**Figure 2:**
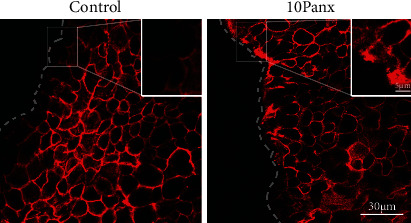
Pannexin1 mediates corneal epithelial sheet migration. Representative images of B6 control corneas (in the presence of 10Panx or a scrambled control (control) peptide) wounded, incubated for 2 hrs in medium, fixed, and then stained with rhodamine phalloidin. The inset highlights the differences in actin at the leading edge and demonstrates coalescence of actin in the 10Panx treated corneas. Bar equals 30 microns. *N* is a minimum of 3 independent experiments.

Since the localization of pannexin1 in B6 control and DiO epithelium at 2 hrs postdebridement differed in both intensity and number of cells displaying enhanced staining near the leading edge, we investigated the role that pannexin1 plays in migration and cell-cell communication. To examine this, B6 control corneas were incubated with either 10Panx (a specific peptide to inhibit pannexin1) or a scrambled control peptide and examined 2 hrs after epithelial debridement (Figure 2). When the corneas were incubated in medium containing the scrambled peptide, the F-actin displayed typical actin cytoskeletal reorganization at the leading edge of the wound and was minimal at the side of the cells facing the wound. In the presence of 10Panx, F-actin is coalesced at the leading edge, and there is an irregularity in cell shape near the wound.

**Figure 3:**
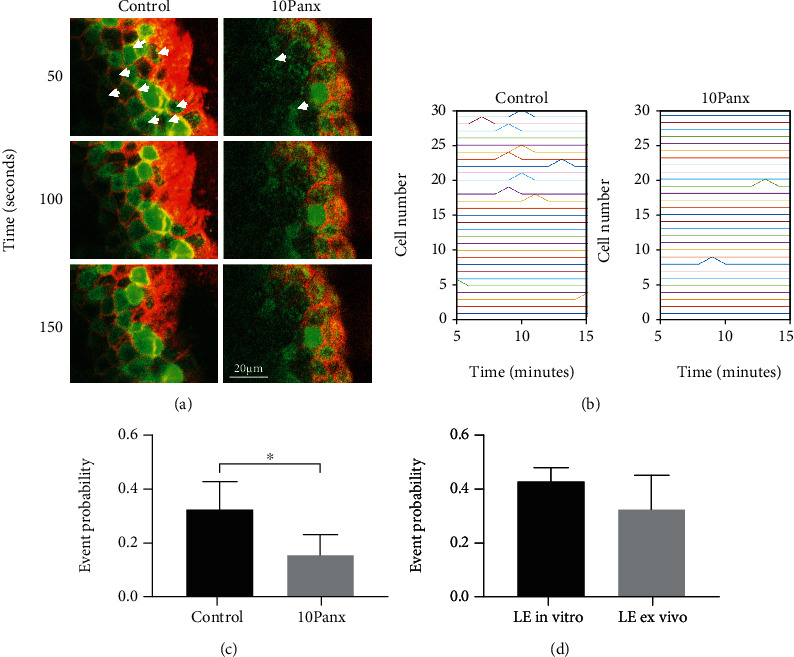
Pannexin1 mediates communication between corneal epithelial cells. (a) Representative images of B6 control corneas incubated in the presence of 10Panx or a scrambled control (control) peptide and then labelled with Cal-520 AM and CellMask Deep Red for imaging. Representative images of the Ca^2+^ wave in basal epithelial cells after injury: 50, 100, and 150 seconds. Arrowheads mark cells to follow over time. (b) MATLAB event charts showing the Ca^2+^ mobilization events detected over time. (c) Event probability values comparing corneas preincubated in the presence of either 10Panx or scrambled control (control) peptide followed by an epithelial scratch injury. (d) Event probability values comparing the wound response near the leading edge of the corneal epithelium ex vivo to in vitro following a scratch wound. Data are expressed as mean ± SEM and were analyzed with a one-way ANOVA with the Tukey's multiple comparison test. *p* < 0.05. *N* is a minimum of 3 independent experiments.

To examine the role of pannexin1 in cell-cell communication, Ca^2+^ mobilization live-cell experiments were performed on corneal epithelium after injury. B6 control corneas were loaded with Cal-520 AM, a calcium indicator that has a long half-life and is more intense than Fluo-3 AM, and counter-stained with CellMask Deep Red, a plasma membrane stain that allows for visualization of corneal epithelium [18]. The latter dye allowed us to identify the basal cells while imaging, as the corneal epithelium is multilayered. The corneas were incubated with either the scrambled control peptide (control) or 10Panx, and events were recorded over time (Figure 3(a)). The images were exported into a MATLAB script [18], and MATLAB-detected events were identified as fluorescence events that were greater than a 50% threshold of the maximum normalized fluorescence signal. Cells were identified using coordinates from the image study video, and representative event charts were generated for each condition (Figure 3(b)). When pannexin1 was inhibited, the MATLAB-detected events were dramatically reduced (Figures 3(a) and 3(b)). These results indicate that cell-cell communication requires the pannexin1 channel. In Figure 3(c), we analyzed the role of pannexin1 on communication between cells in the corneal epithelium and demonstrated that there was a significant decrease (*p* < 0.05) in the likelihood (or event probability) that cells would signal one another when pannexin1 was inhibited with 10Panx. These results support our hypothesis that pannexin1 channels play a crucial role in Ca^2+^ mobilization between corneal epithelial cells and that most of the communication occurs at the leading edge. This study allowed us to examine the dynamic communication that occurred after injury in the basal cells at the leading edge. Future experiments will be performed to examine communication between the different layers of the epithelium and regions of the cornea. The coordination of the response observed in Figure 3 may be explained in part by the fact that pannexin1, a channel protein, transports ATP out of the cell and may couple the cells at the leading edge [28]. These data are supported by earlier studies in other cell systems that suggest the presence of a feed-forward system where ATP moves through pannexin1 channels and activates P2X7 receptors [24]. Future studies will examine levels of ATP released when pannexin1 channels are inhibited with 10Panx in obese or diabetic tissue. However, the results from the ex vivo corneal epithelium experiments suggest that the release of ATP along the wound margin was inhibited, thereby removing the ATP gradient that Junger demonstrated in neutrophils [29].

In addition, we wanted to assess if the events that occurred after injury in our ex vivo results were similar to those that we detected from our in vitro experiments. To do this, we compared the probability that cells near the leading edge would communicate to another cell in both systems, and we found that there was no significant difference (Figure 3(d)). The consistency is intriguing, as the in vitro cultures represent a single epithelial layer, while the ex vivo imaging is of basal corneal epithelial cells from an intact cornea.

### 3.3. Interaction of Pannexin1 and P2X7

The resulting intercellular Ca^2+^ mobilization and actin reorganization of diabetic corneal epithelium indicate an intricate play in communication between the 2 channel proteins, pannexin1 and P2X7. Upon analysis of the proposed pannexin1 signalome [30], we hypothesized that there were significant changes in the interaction between P2X7 and pannexin1 in the pretype 2 diabetic corneal epithelial model (DiO) compared to the B6 control. This change in association may underlie the delayed and disorganized wound healing observed in diabetic corneal models. Although previous immunoprecipitation experiments demonstrated an association between pannexin1 and P2X7 in cultured epithelium [18], these experiments did not address the localization of the associations in unwounded and wounded corneal epithelium, or how the association was altered in a diabetic cornea. To address this, we performed PLA on both unwounded corneas and corneas at 2 hrs after injury. This time point was chosen because previous experiments have shown that there was a significant difference in localization of P2X7 and pannexin1 between the 2 murine models. The amplification process that ultimately generates a visible signal will only occur if the two antibodies are within 40 nm of one another. This technique was used to detect P2X7/pannexin1 interaction throughout the tissue, and specifically, at the region adjacent and distal to the wound.

**Figure 4:**
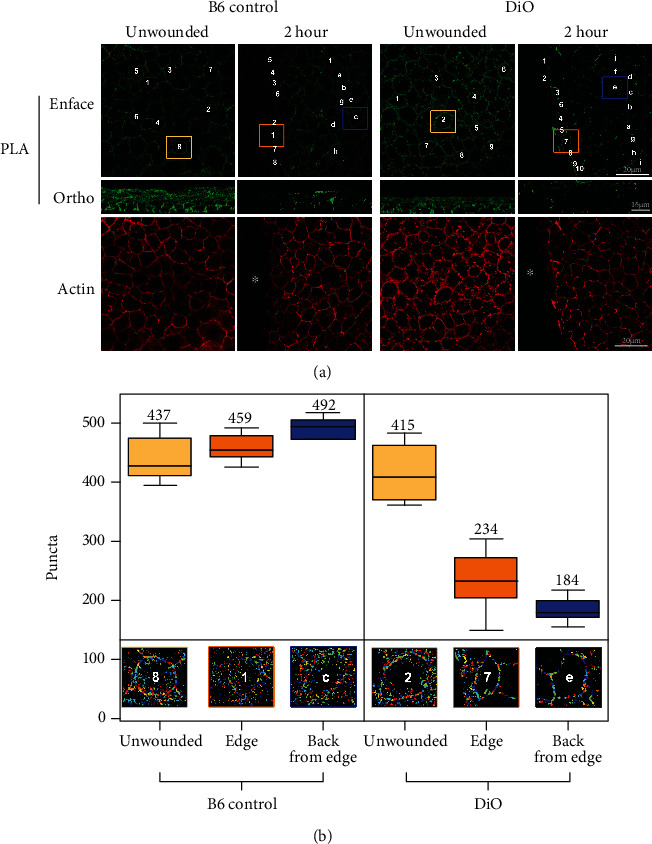
Association of pannexin1 and P2X7 in unwounded corneas and 2 hrs after injury in DiO and B6 control corneal epithelium. Proximity ligation assays were performed using antibodies to P2X7 and pannexin1. (a) Representative enface and orthogonal (ortho) images of B6 control and DiO corneas are shown. The green color displays puncta of association. Corneal epithelium was counter stained with rhodamine phalloidin (asterisk marks wound area). The numbers and letters mark the individual cells that were analyzed using CellProfiler near the wound and back from the wound. The boxes indicate the representative individual cells that are shown in (b) after PLA analysis with CellProfiler. Bar equals 20 microns (PLA enface and actin) or 15 microns (PLA ortho). (b) Analysis of overlapping puncta for each condition (unwounded, leading edge, back from leading edge) was performed and a box plot drawn using R. The mean puncta for each region are placed above the box. Mean ± SEM are plotted, and two-way ANOVA with Tukey's multiple comparison of means was performed. *p* < 0.05. *N* is a minimum of 3 independent experiments.

Optical images were collected from the basal through to the apical layer of the epithelium and presented as enface (basal layer) and orthogonal (full thickness) views (Figure 4(a)). All imaging was performed at the same laser gain and intensity settings that were established through examination of the negative controls and subsequent analysis of overlapping pixel points (see Methods). Counterstaining of the epithelium with rhodamine phalloidin (Figure 4(a), actin) was performed to improve visibility of the tissue. In the unwounded B6 control epithelium, P2X7 and pannexin1 displayed strong colocalization along cell borders (Figure 4(a)). In the orthogonal image, there were many puncta in both the apical and basal cells. In the DiO enface and orthogonal sections, the P2X7 and pannexin1 association was similar to the B6 control (Figure 4(a)). Two hours after injury, there was a distinct change between the association in B6 control and DiO tissue. In the B6 control, there was intense staining in the enface and orthogonal images. There were significantly more PLA punctae in most apical cells than there were in the basal cells, and there appeared to be diminished association at the leading edge (Figure 4(a), ortho). In the DiO tissue, after injury, there was a “line” of fluorescence at the leading edge, yet substantially fewer colocalized punctae back from the wound. As with the B6 controls, the DiO corneas displayed intense staining of apical epithelial cells; however, there was only minimal association in the basal cells. This qualitative data was exported into ImageJ for preliminary processing and then analyzed and quantified using the CellProfiler software. We optimized the CellProfiler pipeline to identify and enhance punctae of the appropriate size, as determined by preliminary analysis in ImageJ. Cells were analyzed individually to ensure optimal detection of PLA puncta. Several cells both at and back from the leading edge of the wound were chosen for analysis to ensure statistical significance. The mean number of puncta for unwounded, edge of wound, and back from edge of wound conditions was calculated for both B6 control and DiO samples. Two-way ANOVA with Tukey's multiple comparison of means was performed. In the unwounded tissue, there was no statistically significant difference in the number of puncta per micron between B6 control and DiO tissue (*p* = 0.779) (Figure 4(b)). Furthermore, after injury in the B6 control, the number of puncta did not change significantly (*p* = 0.792 for the edge and *p* = 0.0238 back from the edge). In contrast, in the DiO tissue, there was a significant decrease in puncta both near and distal to the leading edge (*p* = 0.0000001 for both) (Figure 4(b)). We predict that the lack of association may be an underlying reason for the aberrant migration in DiO corneas after wounding and have preliminary data that the diabetic cells migrate, but the directionality is impaired. In the future, Ca^2+^ mobilization and migration experiments will be performed on diabetic corneal epithelium to determine the downstream effects of decreased cell-cell communication.

These are the first studies to use quantitative image processing of tissues to examine cell-cell communication and to correlate this data with the association of 2 ion channels. This model may be used to identify therapeutic targets and test strategies in the cornea and in other tissues to modulate the treatment and prevent disease progression.

## 4. Conclusions

In summary, our study provides comprehensive data demonstrating that pannexin1 has a different localization in B6 control and an obese pretype 2 diabetic model (DiO). In addition, pannexin1 inhibition impairs cell-cell communication of corneal epithelium, indicating that pannexin1 plays a critical role in wound repair. In the pretype 2 diabetic corneal epithelium, the association of P2X7 and pannexin1 was negligible, indicating that the interaction of the proteins was lost, thereby, disrupting the interactome and subsequent downstream signaling.

## Data Availability

The immunohistochemical and signaling data used to support the findings of this study are included within the article. Previously reported Ca^2+^ mobilization data was used to support this study and is available at doi: 10.1371/journal.pone.0213422. These prior studies and datasets are cited at relevant places within the text and referenced as Lee et al. [18].
